# Left ventricular geometry characteristics and clinical outcomes in hemodialysis patients with heart failure with preserved ejection fraction

**DOI:** 10.1186/s12872-024-03985-x

**Published:** 2024-06-27

**Authors:** Yi Zhang, Xiaofei Guo, Sijiao Chen, Yin Wang, Jingjing Li, Xiaofeng Sun, Xiaomei Huang

**Affiliations:** 1 Department of Ultrasound, Tongji Medical College, The Central Hospital of Wuhan, Huazhong University of Science and Technology, Wuhan, 430014 China; 2 Department of Nephrology, Tongji Medical College, The Central Hospital of Wuhan, Huazhong University of Science and Technology, Wuhan, 430014 China

**Keywords:** All-causemortality, Cardiovascular events, Hemodialysis, Left ventricular hypertrophy, Left ventricular geometry, Left ventricular systolic function

## Abstract

**Background:**

The relationships among left heart remodeling, cardiac function, and cardiovascular events (CEs) in patients with heart failure (HF) with preserved ejection fraction (HFpEF) undergoing maintenance hemodialysis (MHD) remain unclear. We evaluated the echocardiographic characteristics and clinical outcomes of such patients with diverse left ventricular geometric (LVG) configurations.

**Methods:**

Overall, 210 patients with HFpEF undergoing MHD (cases) and 60 healthy controls were enrolled. Cases were divided into four subgroups based on LVG and were followed up for three years. The primary outcomes were the first CEs and all-cause mortality.

**Results:**

Left ventricular ejection fraction (LVEF) and right ventricular systolic function did significantly differ between cases and controls, whereas echocardiographic parameters of cardiac structure, diastolic function, and left ventricular global longitudinal strain (LVGLS) differed significantly. The proportion of cases with left ventricular hypertrophy (LVH) was 67.1%. In addition, 2.38%, 21.90%, 12.86%, and 62.86% of cases presented with normal geometry (NG), concentric remodeling (CR), eccentric hypertrophy (EH), and concentric hypertrophy (CH), respectively. The left atrial diameter (LAD) was the largest and cardiac output index was the lowest in the EH subgroup. The score of Acute Dialysis Quality Initiative Workgroup (ADQI) HF class was worse in the EH subgroup than in other subgroups at baseline. The proportions of cases free of adverse CEs in the EH subgroup at 12, 24, and 36 months were 40.2%, 14.8%, and 0%, respectively, and the survival rates were 85.2%, 29.6%, 3.7%, respectively, which were significantly lower than those in other subgroups. Multivariate Cox regression revealed that age, TNI (Troponin I), EH, left ventricular mass index (LVMI), age and EH configuration were independent risk factors for adverse CEs and all-cause mortality in the cases.

**Conclusion:**

Most patients with HFpEF receiving MHD have LVH and diastolic dysfunction. Among the four LVGs, patients with HFpEF undergoing MHD who exhibited EH had the highest risk of adverse CEs and all-cause mortality.

**Supplementary Information:**

The online version contains supplementary material available at 10.1186/s12872-024-03985-x.

## Background

Adverse cardiovascular events (CEs) are the main cause of mortality in patients undergoing maintenance hemodialysis (MHD) owing to concomitant cardiac hypertrophy and/or changes in left ventricular geometry (LVG). Left ventricular hypertrophy (LVH) in these patients is associated with shorter survival without CEs and a higher risk of heart failure (HF) [1–3]. HF is characterized by increased thickness of the left ventricular wall, an enlargement of the left ventricular chamber, or both [3]. The Framingham heart study classified LVG into four types based on left ventricular mass (LVM) and relative wall thickness (RWT): concentric hypertrophy (CH), increased LVM and RWT; eccentric hypertrophy (EH), increased LVM and normal RWT; concentric remodeling (CR), normal LVM and increased RWT; and normal geometry (NG), normal LVM and normal RWT [4]. Previous studies have shown that LVM, traditional cardiovascular risk factors, and different LVGs can affect patient prognosis [5, 6]. However, previous studies have reported diverse prognostic outcomes; for example, some authors have proposed that CH-linked increased peripheral resistance, decreased cardiac output, and decreased plasma volume are associated with worse prognosis. Meanwhile, other authors have suggested that EH, which is linked to increased plasma volume, among others, may lead to poor prognosis [7]. The prevalence heart failure (HF) with preserved ejection fraction (HFpEF) in patients undergoing MHD is 40–76.5% [8]. Previous studies have described the mechanical and clinical causes of worsening LVG associated with chronic kidney disease (CKD) [9, 10]. However, the relationships among left heart remodeling, cardiac diastolic and systolic function, and CEs in patients with HFpEF receiving MHD remain unclear [11]. We aimed to describe the echocardiographic characteristics and LVG distribution of patients with HFpEF undergoing MHD and analyze the association of these characteristics with the incidence of CEs and all-cause mortality.

## Methods

This was a single-center, retrospective study. Patients receiving MHD in Wuhan Central Hospital between August 2020 and March 2021 were screened for inclusion. Overall, 210 patients met the inclusion and exclusion criteria and were categorized into four subgroups based on LVG. Sixty healthy controls were matched based on age, sex, and body mass index during the same period. The controls had no history of cardiovascular disease, diabetes, or kidney disease, with normal 12-lead electrocardiogram and transthoracic echocardiography findings. This study was approved by the ethics committee of Wuhan Central Hospital (approval Document: 2016 Medical Research No. 03 and Hospital-Heng-Lun letter-2021 [9]). Informed consent was obtained from each patient, and the study protocol conformed to the ethical guidelines of the 1975 Declaration of Helsinki. The trial was registered with Chinese Clinical Trial Registry (https://www.chictrorg.cn/), registration number ChiCTR2200061199. dated 2022/6–15.

The inclusion criteria were as follows: (1) age > 18 years; (2) hemodialysis vintage ≥ 3 months and willingness to participate in the study; (3) diagnosis of HF with documented ejection fraction (EF) of ≥ 50% based on echocardiography performed within six months before screening; and (4) vascular access for MHD using an arterial venous fistula (AVF).

The exclusion criteria were as follows: (1) refusal to follow medical advice or loss to follow-up; (2) prior left ventricular ejection fraction (LVEF) < 50% detected by echocardiography within six months before screening; (3) HF primarily resulting from precordial hypertrophic obstructive cardiomyopathy, severe heart valve disease (mitral valve lesion/aortic valve lesion), isolated right HF, constrictive pericarditis, or cardiac resynchronization treatment; (4) myocardial infarction diagnosed clinically within three months or undergoing percutaneous coronary intervention or coronary artery bypass surgery combined with malignancy, congenital heart disease, or tuberculosis; and (5) weight gain ≥ 10% of dry weight between dialysis sessions, even with a dialysis frequency of three times per week and total dialysis time ≥ 10 h/week (which repeatedly occurred more than three times within one month of screening). Venous blood samples were collected in the morning before hemodialysis with the participants seated. Dialysis inter-blood pressure and weight gain values were the averages of the values recorded during the month of study enrollment.

According to the current guidelines [12], EH was defined as left ventricular mass index (LVMI) > 95 g/m^2^ for women and > 115 g/m^2^ for men with an RWT ≤ 0.42; CH was defined as LVMI > 95 g/m^2^ for women and > 115 g/m^2^ for men with RWT > 0.42; CR was defined as LVMI ≤ 95 g/m^2^ for women and ≤ 115 g/m^2^ for men with RWT > 0.42; and NG was defined as LVM ≤ 95 g/m^2^ for women and ≤ 115 g/m^2^ for men with RWT ≤ 0.42.

Adverse CEs were defined as stroke, transient ischemic attack, angina pectoris, acute myocardial infarction, sudden cardiac arrest or death, acute HF, and severe arrhythmia (requiring hospitalization or lasting > 24 h). The study endpoints were any of the CEs or all-cause mortality recorded during the follow-up period. Family members of patients who died outside the hospital were interviewed by telephone to determine the cause of death. The time of the first event was used for survival analysis in patients who suffered multiple CEs.

### Statistical analysis

All statistical analyses were conducted using SPSS 25.0 statistical software (IBM Corp., Chicago, IL, USA). Continuous normally distributed variables were expressed as means and standard deviations, and non-normally distributed variables were expressed as medians and interquartile ranges. The *t*-test and Wilcoxon test were used to compare continuous data, and the *χ*^2^ or Fisher’s exact test was used to compare categorical data. The relationships between echocardiographic parameters and the first CEs or all-cause mortality were analyzed using Kaplan–Meier survival and Cox regression analysis. Pearson’s correlation was used to evaluate the relationship between the log-transformed values of brain natriuretic peptide (BNP) and echocardiography parameters, including LVG, LVEF, and left ventricular global longitudinal strain (LVGLS). All p-values were two-sided, and statistical significance was set at p-value < 0.05.

## Results

### Baseline clinical characteristics and cardiac ultrasound indicators in the control and MHD groups

Overall, 60 participants were included in the normal control group (mean age, 55.7 ± 12.3 years), and 210 were included in the MHD group (mean age, 55.3 ± 9.9 years). The MHD group had higher blood pressure and faster heart rates than the normal control group; higher creatinine, calcium, phosphorus, lipids, and parathyroid hormone levels; and lower average hemoglobin and albumin levels.The left atrium and ventricle were enlarged; the left ventricular volume index (LVEDVi, LVESVi, COi) and LVEF were decreased; interventricular septal thickness (IVST) and left ventricular posterior wall thickness (LVPW) were thickened (all *P* < 0.05); and the RWT and LVMI were increased in the MHD group compared with the normal group. The incidence of LVH was 67.1% (141/210) in the MHD group. LVGLS (*P* < 0.001) and left heart diastolic function indices (E/A, E/e, LAVi) were significantly lower (*P* < 0.05) in the MHD group. Right ventricular systolic function was not significantly different between the groups (RV-FAC%, TAPSE) (Table 1).

**Table 1:** Baseline clinical characteristics and cardiac ultrasound indicators in the control and MHD groups

	Control(*n* = 60)	MHD patients(*n* = 210)	*P*/X^2^
Sex, female, n(%)	30(50%)	104(49.5%)	0.53
Age	55.7 ± 12.3	55.3 ± 9.9	0.84
BMI	22.92 ± 2.52	23.16 ± 3.46	0.62
SBP, mmHg	115.6 ± 7.5	146.22 ± 20.39	<0.01
DBP, mmHg	74.2 ± 5.69	80.67 ± 13.31	<0.01
HR, bpm	68.94 ± 11.42	74.24 ± 13.3	0.01
Hemoglobin, g/L	136.06 ± 15.92	101.06 ± 16.84	<0.01
Alb, g/L	43.1 ± 7.51	40.03 ± 4.57	<0.01
Ca, mmol/L	2.43 ± 0.19	2.26 ± 0.21	<0.01
P, mmol/L	1.09 ± 0.14	1.69 ± 0.52	<0.01
PTH, pg/mL	44.87(40.23–49.50)	200.32(168.28-232.37)	<0.01
hs-CRP, mg/dl	0.14(0.12–0.17)	0.36(0.28–0.44)	0.01
TC, mmol/L	3.86 ± 0.88	3.83 ± 1.13	0.86
Triglyceride, mmol/L^a^	1.27 ± 0.31	1.54 ± 1.21	0.09
LDL, mmol/L	1.93 ± 0.8	2.19 ± 0.74	0.02
HDL, mmol/L	1.37 ± 0.21	1.1 ± 0.39	<0.01
TNI, ng/L	0.01 ± 0.03	0.04 ± 0.21	0.35
BNP*	2.33 ± 0.59	2.73 ± 0.66	<0.01
UF, L		2.74 ± 0.76	<0.01
Scr, umol/L	54.47(58.56–7.34)	690.2(635.76-744.64)	<0.01
Echocardiographic characteristics			
LAD, cm	3.41 ± 0.47	3.78 ± 0.59	<0.01
LVEDd, cm	4.31 ± 0.55	4.53 ± 0.57	<0.01
RAD, cm	3.37 ± 0.47	3.34 ± 0.58	0.705
RVD, cm	3.22 ± 0.47	3.25 ± 0.49	0.671
IVST, cm	1.12 ± 0.15	1.21 ± 0.19	<0.01
LVPW, cm	1.08 ± 0.14	1.16 ± 0.16	0.002
LVEDVi, ml/ m^2^	48.85 ± 11.99	52.83 ± 18.33	0.12
LVESVi, ml/ m^2^	17.78 ± 5.77	19.46 ± 8.8	0.11
COi, L/min/ m^2^	2.23 ± 0.54	2.4 ± 0.74	0.11
RWT	0.45 ± 0.08	0.51 ± 0.09	<0.01
LVMi, g/m^2^	97.8 ± 13.23	132.42 ± 36.23	<0.01
Systolic function			
LVEF,%	64.18 ± 5.4	63.76 ± 5.24	0.59
LVGLS,%	-19.72 ± 2.97	-16.46 ± 3.43	<0.01
LVGCS,%	-23.76 ± 5.02	-21.95 ± 5.55	0.03
LVGRS,%	-41.98 ± 6.41	-31.85 ± 16.73	<0.01
Diastolic function			
E/A ratio	1.03 ± 0.38	0.87 ± 0.37	<0.01
E/e’ ratio (septal)	8.72 ± 2.63	12.72 ± 5.08	<0.01
LAVi, ml/m^2^	24.08 ± 7.18	31.01 ± 13.63	<0.01
Right ventricular function			
FAC,%	49.78 ± 9.02	49.03 ± 8.01	0.53
TAPSE, cm	2.15 ± 0.27	2.16 ± 0.36	0.83
RVGLS,%	-26.31 ± 5.32	-26.98 ± 6.35	0.46

Values are expressed as n, mean ± SD, n (%),or median (interquartile range), unless otherwise indicated

Abbreviations: Alb, albumin; ADQI, The Acute Dialysis Quality Initiative Workgroup; AVF, arteriovenous fistula; BMI, body mass index; BNP*,BNP-After logarithmic conversion; Ca, calcium; COi, cardiac output index; DBP, diastolic blood pressure; E/A, peak early diastolic transmitral flow velocity/peak late diastolic transmitral flow velocity; E/e′,peak early diastolic transmitral flow velocity/peak early diastolic mitral annular tissue velocity; HR, heart rate; HDL, high-density lipoprotein cholesterol; hs-CRP, high sensitivity C-reactive protein; LAD, left atrial diameter; LDL, low-density lipoprotein cholesterol; LVEDd, left ventricular end-diastolic diameters; IVST, interventricular septal thickness; LVPW, left ventricular posterior wall thickness; LVEDVi, left ventricular end-diastolic volume index; LVMI, left ventricular mass index; LVEF, left ventricular ejection fraction; LVGLS, left ventricular global longitudinal strain; LVGCS, left ventricular global circumferential strain; LVGRS, left ventricular global radial strain; LVESVi, left ventricular end-systolic volume index; LAVi, left atrial volume index; P,phosphorus; PTH, parathyroid hormone; SBP, systolic blood pressure; RVFAC, right ventricular fractional area change; RAD, right atrial diameter; RASI, renin-angiotensin aldosterone system inhibitor; RVD, right ventricular diameter; RWT, relative wall thickness; RVGLS, right ventricular global longitudinal strain; TAPSE, tricuspid annular plane systolic excursio; TC, total cholesterol; TNI, troponin I; UF, ultrafiltration

### Baseline clinical characteristics and cardiac ultrasound indicators in the MHD subgroups

No differences were observed in the primary disease, medications, dialysis vintage, and AVF blood flow in patients receiving MHD with different LVGs, whereas PTH and BNP levels were higher in the CH subgroup than in the other subgroups.

Echocardiography parameters significantly differed in different LVG subgroups of patients undergoing MHD. The CH subgroup accounted for the largest percentage in patients undergoing MHD, reaching 62.86% (132/210). LVMI (CH > EH > NG > CR) and volume loading (LVEDVi, LVESVi, and COi) (CH > CR > EH > NG) were the highest, diastolic function (E/A, E/e’ ratio, LAVi) (CH > CR > EH > NG) was the worst, and LVGLS (CH < CR < EH < NG) was the lowest in the CH subgroup than in other LVG subgroups. The LAD was the largest in the EH subgroup (*P* = 0.0014) (Table 2).

**Table 2:** Baseline clinical characteristics and cardiac ultrasound indices in patients with MHD with different LVGs

.	NG(*n* = 5)	CR(*n* = 46)	CH(*n* = 132)	EH(*n* = 27)	*P*/X^2^
Sex, female, n(%)	0(0%)	19(41.3%)	75(56.8%)	10(37%)	0.04
Age	52.4 ± 10.8	54.7 ± 11.5	55.3 ± 9.9	57. 2 ± 7.0	0.655
BMI	22.97 ± 1.7	23.45 ± 3.25	22.78 ± 3.59	24.54 ± 3.10	0.101
SBP, mmHg	154.4 ± 16.34	140.34 ± 18.43	146.63 ± 20.28	152.7 ± 22.82	0.059
DBP, mmHg	79.4 ± 12.21	80.43 ± 12.58	80.75 ± 13.4	80.88 ± 14.93	0.995
HR, bpm	83.11 ± 17.11	75.71 ± 15.57	73.63 ± 12.53	73.09 ± 11.95	0.354
Hemoglobin, g/L	101.6 ± 10.16	98.43 ± 18.66	101.14 ± 16.55	105.03 ± 15.92	0.455
Alb, g/L	41.51 ± 1.11	39.86 ± 5.05	39.81 ± 4.57	41.08 ± 4.07	0.513
Ca, mmol/L	2.28 ± 0.18	2.26 ± 0.16	2.28 ± 0.21	2.17 ± 0.25	0.098
P, mmol/L	1.73 ± 0.54	1.64 ± 0.48	1.7 ± 0.5	1.74 ± 0.68	0.86
PTH, pg/mL	137.14(56.2-249.91)	121.86(81.48-162.23)	232.07(185.93-278.79)	190.53(118.27-262.79)	0.047
hs-CRP, mg/dl	0.27(0.021–0.52)	0.36(0.15–0.58)	0.32(0.24–0.40)	0.57(0.26–0.88)	0.238
TC, mmol/L	3.03 ± 0.74	3.92 ± 1.14	3.79 ± 1.14	4.07 ± 1.09	0.25
Triglyceride, mmol/La	0.89 ± 0.42	1.32 ± 0.91	1.62 ± 1.23	1.62 ± 1.53	0.298
LDL, mmol/L	1.87 ± 0.72	2.16 ± 0.87	2.25 ± 0.66	2.03 ± 0.88	0.386
HDL, mmol/L	0.9 ± 0.38	1.22 ± 0.41	1.07 ± 0.37	1.14 ± 0.42	0.088
TNI, ng/L	0 ± 0	0.06 ± 0.31	0.04 ± 0.19	0.02 ± 0.04	0.836
BNP*	1.75 ± 0.2	2.55 ± 0.41	2.86 ± 0.67	2.60 ± 0.74	0.027
UF, L	2.8 1 ± 0.85	2.91 ± 0.56	2.70 ± 0.82	2.72 ± 0.75	0.2
Scr, umol/L	479.6(255.5–1189)	471(355–586)	787(724–849)	631(472–789)	<0.01
ADQI class for HF-n (%)					
2R-2NR	4(1.90)	18(8.57)	66(31.43)	16(7.62)	0.133
3R-3NR	1(0.48)	7(3.33)	35(16.67)	8(3.81)	0.091
4R-4NR	0	0(0)	10(4.76)	5(2.38)	<0.01
AVF Location					
Fore arm	5	43	126	26	0.126
Upper arm	0	2	6	3	0.333
AVF Blood flow(ml/min)	658.6 ± 58.58	928.39 ± 622.67	866 ± 420.1	740.89 ± 346.60	0.287
Dialysis time (Month)	21.07 ± 13.89	25.41 ± 36.96	41.12 ± 42.82	35.9 ± 39.89	0.088
Basic diseases, n(%)					
Hypertension	4(1.9)	40(19.05)	121(57.62)	20(9.52)	0.145
Diabetes	3(1.43)	11(5.24)	57(27.14)	10(4.76)	0.212
Others	2	2	11	5	0.124
Antihypertensive medicines, n (%)					
RASI	2(0.95)	19(9.05)	111(52.86)	10(4.76)	0.434
CCB	4(1.9)	30(14.28)	122(58.09)	11(5.24)	0.111
α-B	0(0)	9(4.28)	48(22.86)	2(0.95)	0.323
BB	3(1.43)	20(9.52)	98(46.67)	3(1.43)	0.498
Echocardiographic characteristics					
LAD, cm	3.51 ± 0.18	3.55 ± 0.52	3.84 ± 0.60	3.90 ± 0.66	0.014
LVEDd, cm	4.60 ± 0.30	4.33 ± 0.58	4.57 ± 0.56	4.65 ± 0.58	0.048
RAD, cm	3.5 ± 0.57	3.25 ± 0.52	3.40 ± 0.61	3.13 ± 0.43	0.087
RVD, cm	3.36 ± 0.55	3.29 ± 0.48	3.22 ± 0.52	3.33 ± 0.28	0.622
IVST, cm	1.04 ± 0.13	1.12 ± 0.17	1.26 ± 0.19	1.16 ± 0.15	<0.01
LVPW, cm	1.02 ± 0.11	1.09 ± 0.18	1.19 ± 0.15	1.11 ± 0.14	<0.01
LVEDVi, ml/ m^2^	32.52 ± 12.04	47.04 ± 15.69	56.86 ± 19.20	46.16 ± 11.66	<0.01
LVESVi, ml/ m^2^	10.79 ± 3.95	16.54 ± 6.69	21.48 ± 9.48	16.16 ± 5.29	<0.01
COi, L/min/ m^2^	1.57 ± 0.45	2.21 ± 0.7	2.55 ± 0.75	2.1 ± 0.53	<0.01
RWT	0.4 ± 0.02	0.52 ± 0.08	0.54 ± 0.08	0.39 ± 0.03	<0.01
LVMi, g/m^2^	104.57 ± 3.39	88.5 ± 14.91	147.94 ± 30.56	136.49 ± 25.68	<0.01
Systolic function					
LVEF,%	66.8 ± 1.09	65.45 ± 4.64	62.83 ± 5.37	64.88 ± 5.04	0.007
LVGLS,%	-19.4 ± 1.94	-16.69 ± 2.73	-16.03 ± 3.15	-17.62 ± 5.18	0.027
LVGCS,%	-23.4 ± 6.02	-23.56 ± 4.52	-21.68 ± 5.71	-20.25 ± 5.86	0.07
LVGRS,%	-43.56 ± 9.49	-32.05 ± 17.45	-31.36 ± 17.72	-31.73 ± 9.92	0.465
Diastolic function					
E/A ratio	0.65 ± 0.14	0.84 ± 0.39	0.92 ± 0.38	0.68 ± 0.16	0.008
E/e’ ratio (septal)	10.09 ± 1.5	10.66 ± 3.8	13.7 ± 5.51	11.94 ± 3.83	0.002
LAVi, ml/m^2^	16.09 ± 2.86	28.76 ± 11.15	33.06 ± 13.81	27.56 ± 15.21	0.007
Right heart function					
FAC,%	44.62 ± 4.2	50.84 ± 5.92	48.44 ± 8.7	49.64 ± 7.74	0.191
TAPSE, cm	2.39 ± 0.25	2.06 ± 0.34	2.19 ± 0.37	2.14 ± 0.27	0.082
RVGLS,%	-25.8 ± 8.28	-28.08 ± 6.18	-27.21 ± 6.4	-24.2 ± 5.5	0.074

Values are expressed as n, mean ± SD, n (%),or median (interquartile range), unless otherwise indicated

Abbreviations: Alb, albumin; ADQI, The Acute Dialysis Quality Initiative Workgroup; AVF, arteriovenous fistula; BMI, body mass index; BB,β-receptor blocker; BNP*,BNP-After logarithmic conversion; Ca, calcium; CCB, calcium channel blocker; COi, cardiac output index; DBP, diastolic blood pressure; E/A, peak early diastolic transmitral flow velocity/peak late diastolic transmitral flow velocity; E/e′,peak early diastolic transmitral flow velocity/peak early diastolic mitral annular tissue velocity; HR, heart rate; HDL, high-density lipoprotein cholesterol; hs-CRP, high sensitivity C-reactive protein; LAD, left atrial diameter; LDL, low-density lipoprotein cholesterol; LVEDd, left ventricular end-diastolic diameters; IVST, interventricular septal thickness; LVPW, left ventricular posterior wall thickness; LVEDVi, left ventricular end-diastolic volume index; LVMI, left ventricular mass index; LVEF, left ventricular ejection fraction; LVGLS, left ventricular global longitudinal strain; LVGCS, left ventricular global circumferential strain; LVGRS, left ventricular global radial strain; LVESVi, left ventricular end-systolic volume index; LAVi, left atrial volume index; P,phosphorus; PTH, parathyroid hormone; SBP, systolic blood pressure; RVFAC, right ventricular fractional area change; RAD, right atrial diameter; RASI, renin-angiotensin aldosterone system inhibitor; RVD, right ventricular diameter; RWT, relative wall thickness; RVGLS, right ventricular global longitudinal strain; TAPSE, tricuspid annular plane systolic excursion; TC, total cholesterol; TNI, troponin I; UF, ultrafiltration;α-B,α-receptor blocker

### Pearson’s correlation analysis in MHD groups

**Fig. 1:**
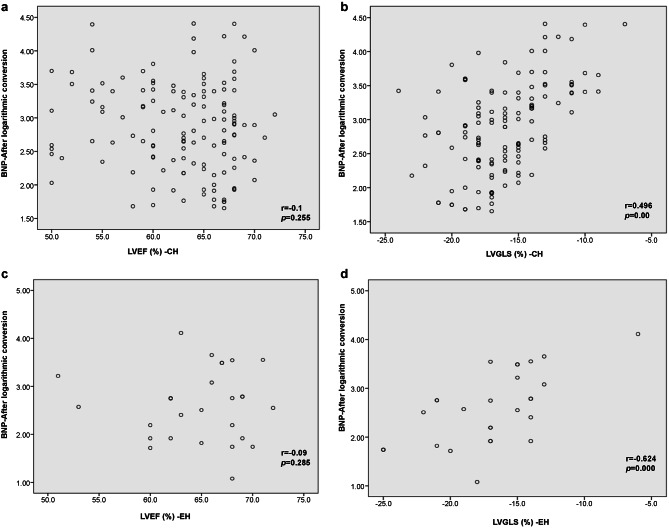
Differences in the correlation between LVGLS, LVEF, and BNP in patients receiving MHD in the CH and EH subgroups. **a**, LVEF in the CH group does not correlate with log-transformed BNP values (*r*=-0.1, *P* = 0.255;); **b**, LVGLS in the CH group significantly correlates with log-transformed BNP values (*r* = 0.496, *P* = 0.000); **c**, LVEF in the EH group does not correlate with log-transformed BNP values (*r*=-0.09, *P* = 0.285); **d**, LVGLS in the EH group significantly correlates with log-transformed BNP values (*r*=0.624, *P*<0.001). LVGLS: left ventricular global longitudinal strain; LVEF: left ventricular ejection fraction; BNP: brain natriuretic peptide; MHD: maintenance hemodialysis; CH: concentric hypertrophy; EH: eccentric hypertrophy

There was a significant difference in BNP between the different LVG subgroups (*P* = 0.027). In the CH and EH subgroups, log-transformed BNP was significantly associated with LVGLS (r_CH_=0.496, *P* = 0.000; r_EH_=0.624, *P* <0.001), but not with LVEF (r_CH_=-0.1, *P* = 0.255; r_EH_=-0.09, *P* = 0.285) (Fig. 1).

### Kaplan–Meier survival analysis for CEs and all-cause mortality among different LVG subgroups in patients undergoing MHD

**Fig. 2:**
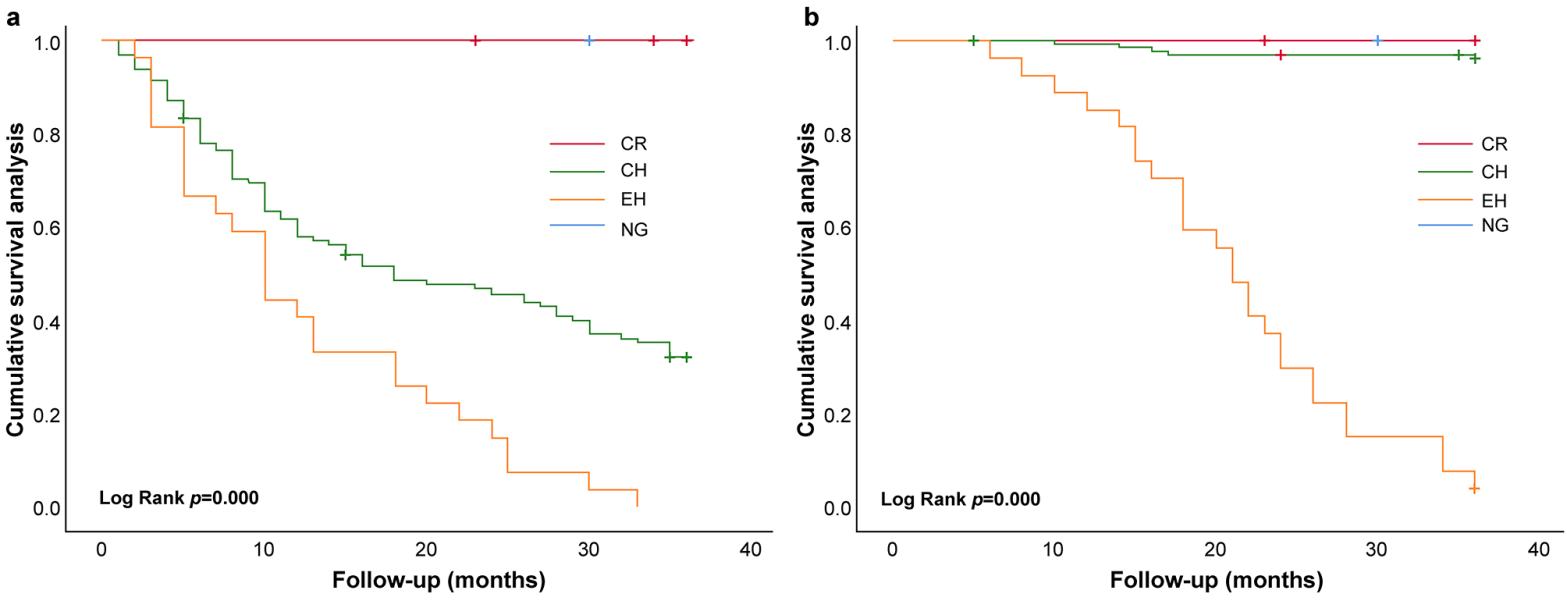
Kaplan–Meier survival curves for cardiovascular event-free and all-cause mortality in patients undergoing MHD with different LVG conformations. **a**, Kaplan–Meier survival curves for patients receiving MHD with different LVG free of CEs (log-rank, *P* = 0.000); **b**, Kaplan–Meier survival curves for patients receiving MHD with different LVG and all-cause mortality (log-rank, *P* = 0.000). MHD: maintenance hemodialysis; LVG: left ventricular geometry

The proportions of patients in the EH subgroup, free of adverse CEs at 12, 24, and 36 months were 40.2%, 14.8%, and 0%, respectively, and the survival rates were 85.2%, 29.6%, and 3.7%, respectively, which were significantly lower than those in the other LVG subgroups (log-rank, *P* = 0.000; Fig. 2).

### Multivariate Cox regression analysis of risk factors for CEs and all-cause mortality among different LVG subgroups in patients undergoing MHD

BNP, LVG, and LVMI were independent risk factors for the first CEs and all-cause mortality, after adjusting for sex, diabetes, and age. Among the LVG subgroups, the EH subgroup had the highest risk for CEs (hazard ratio (HR) = 1.459, 95% confidence intervals (CI) 1.031–2.770, *P* = 0.003) and all-cause mortality (HR = 1.013, 95%CI 1.002–2.201, *P* = 0.000). EF, LVGLS, and E/e’ were not independent risk factors for either the first CEs or all-cause mortality (Table 3).

**Table 3:** Multivariate Cox regression model of risk factors for heart failure-related hospitalization in patients undergoing MHD

Factors	Cardiovascular events				All cause mortality		
	HR	95% CI	*p*		HR	95% CI	*p*
Age, year	1.041	1.016–1.065	0.001		1.037	1.009–1.073	0.003
TNI	3.334	1.421–7.831	0.006		2.644	0.630-60.235	0.202
BNP*	1.031	0.73–1.456	0.864		1.090	0.223–1.897	0.189
LV geometry			0.033				0.000
NG		ref				ref	
CR	1.001	0.980–1.002	0.965		1.001	0.963–1.003	0.977
CH	1.032	0.763–1.224	0.890		1.022	0.861–1.320	0.932
EH	1.459	1.031–2.770	0.003		1.013	1.002–2.201	0.000
EF(%)	0.995	0.961–1.031	0.790		0.939	0.906–1.128	0.458
LVMI	1.026	1.019–1.033	0.000		1.023	1.007–1.041	0.012
LVGLS(%)	1.012	0.956–1.070	0.691		0.970	0.877–1.074	0.682
E/e’	1.008	0.972–1.046	0.664		1.068	0.974–1.172	0.164

Abbreviations: BNP*,brain natriuretic peptide-After logarithmic conversion; LVMI, left ventricular mass index; LVGLS, left ventricular global longitudinal strain; LVEF, left ventricular ejection fraction; E/e’,peak early diastolic transmitral flow velocity/peak early diastolic mitral annular tissue velocity; TNI, troponin

## Discussion

LVH is common in patients undergoing MHD and accounted for 67.1% of cases in this study. Among all LVGs, 62.86% were CH, 21.90% were CR, 12.86% were EH, and 2.38% were NG. LVH is even higher than those of Zhao et al. (LVH: 61.1%) and Paoletti (LVH: 56.0%) [7, 13]. In this study, multivariate Cox analysis showed that traditional risk factors, age, and LVMI remained independent risk factors for CEs and all-cause mortality [14]. Both hemodynamic and non-hemodynamic factors can lead to LVH and LVG changes in patients undergoing MHD [15]. LVH is a complex phenotype that predicts adverse cardiovascular outcomes, rather than serving as an adaptive response [15]. Among the LVG subgroups, the EH subgroup had the highest risk for CEs and all-cause mortality. Hypertrophic myocardium exhibits fibrosis, changes in coronary circulation, and myocardial cell apoptosis, which can result in HF, myocardial ischemia, and arrhythmias. However, the relationship between EH and the sudden death of patients with MHD remains unclear. Some authors [16] proposed that other traditional risk factors (i.e., hypercholesterolemia, hypertension, and obesity) and chronic activation of sympathetic nerves and the renin-angiotensin-aldosterone system may lead to sodium and fluid retention and myocardial fibrosis. Furthermore, non-traditional risk factors such as inflammation, oxidative stress, endothelial dysfunction, and abnormal chronic kidney disease-related mineral bone disease seem to play a role.

In this study, the EH group had the largest LAD (*P* = 0.014). The atrial wall contains various cell types including fibroblasts, adipocytes, immune cells, blood vessels, and non-cellular components, such as collagen fibers, in addition to atrial muscle cells. Therefore, LAD enlargement may result from increased atrial muscle cell volume (hypertrophy), total cell count, or extracellular collagen (fibrosis). LAD can lead to left atrial systolic dysfunction and increase the incidences of arrhythmia and atrial fibrillation, resulting in pressure increases, affecting venous return, and disrupting left ventricular function and filling pressure [5]. Any increase in left ventricular filling pressure may increase ventricular hardness, which is sensitive to changes in cardiac load. Decreased filling pressures and preload during dialysis result in decreased stroke output. However, after left ventricular myocardial remodeling, myocardial mechanics are altered and cannot be compensated for by increasing the contractile force. Decreased cardiac output and hypotension can occur when the heart rate cannot increase to compensate for the decrease in stroke output, leading to thickening and stiffness of the myocardium, decreased coronary artery blood flow, and subsequent deterioration of the myocardial structure [ [17, 21]]. This explains why the CH subgroup presented with poor systolic and diastolic function and the highest cardiac output, whereas the EH group presented with the lowest cardiac output, resulting in a high risk of CEs and all-cause mortality in this subgroup. Arrhythmias, atrial fibrillation, myocardial fibrosis, decreased cardiac output, and decreased coronary perfusion may all be associated with MHD, particularly in patients with EH [9, 18].

LVGLS was significantly lower in patients receiving MHD than in normal controls, whereas the LVEFs were all > 60% and were not statistically different. LVEF reflects cardiac output percentage, considering the Frank–Starling relationship. However, it does not accurately reflect the presence or absence of myocardium dysfunction and is affected by ventricular loading (particularly afterload), which does not allow for early and accurate evaluation of left heart systolic function in patients with MHD in the presence of myocardial hypertrophy and altered myocardial conformation. Particularly, patients with LVH with myocardial insufficiency but small LV chambers retain LVEF in the presence of impaired LVGLS. In this study, diastolic function was significantly reduced in patients with MHD compared with normal controls. Stassen et al. concluded that progressive deterioration of interstitial myocardial fibrosis after LV remodeling increases ventricular wall stiffness, leading to reduced compliance, which severely impacts diastolic function and systolic function thereafter [19]. LVEF values constituted the metrics used to evaluate systolic function in the abovementioned studies. Other studies used strain metrics, including LVGLS, to evaluate systolic function, and found that systolic function in patients with MHD is synchronized with diastolic function reduction [20], in line with the findings of our study. Decreased myocardial systolic function begins in the longitudinal subendocardial myocardium, and LVGLS primarily reflects a contraction in the longitudinal myocardial layer; therefore, LVGLS may provide early detection of left heart systolic dysfunction. LVGLS has some limitations, such as susceptibility to load [21], and its reduction may be associated with other factors, including hypertension, male sex, and smoking [22]. Nevertheless, LVGLS combined with multiple other parameters of systolic function, hemodynamic loading states, and LV remodeling allows for early identification of patients at high risk for progressive MHD.

In this study, the correlation analysis showed that log-transformed BNP was not correlated with LVEF in either the CH or EH subgroup, but LVGLS. There was no correlation between the log-transformed BNP and LVGLS and LVEF in both the NG and CR subgroups. BNP is a biomarker associated with prognosis in patients with HFpEF. Although it is affected by factors such as reduced glomerular clearance, increased myocardial wall relaxation, and clearance by dialysis [30], elevated BNP levels in MHD patients are associated with increased risks of CEs and all-cause death [31]. After adjusting the diagnostic threshold according to renal function, BNP can help to identify patients at a high risk of HF [19, 23]. The correlation between LVGLS and log-transformed BNP in the CH and EH groups indicated an increased risk of CEs and death in the hypertrophic remodeling group, compared to that in the other subgroups.

In this study, LVGLS in patients undergoing MHD decreased. However, right ventricular function and LVEF did not significantly decrease, which can be attributed to the fact that all included patients had LVEF > 50% and strong compensatory function of the pulmonary circulation. Current research shows that the establishment of vascular access may affect the cardiac function or clinical outcomes of patients undergoing MHD [24]. We excluded patients with arteriovenous grafts or other vascular access to reduce the effects of confounding factors.

### Limitations

This study had some limitations. All echocardiograms were performed at rest, limiting the ability to assess the relationship between LVGLS and impaired performance during exercise, an important hallmark of HFpEF [25]. Another limitation is the single-center design and small sample size. Nevertheless, preliminary conclusions that the EH subgroup was at a higher risk of CEs and all-cause mortality among the LVG subgroups and that LVGLS combined with LVG could identify high-risk patients receiving dialysis can be deduced from this study.

## Conclusions

LVH is common among patients undergoing MHD. The CH subgroup accounted for the highest proportion of LVG in patients undergoing MHD, while the EH subgroup showed the highest risk of CEs and all-cause mortality among patients receiving MHD. This effect may account for the clinical heterogeneity of cardiac phenotypes in patients with HFpEF. LVG characteristics and LVGLS need to be combined to evaluate cardiac function in patients with HFpEF with MHD, and patients with MHD presenting as EH configuration should get more attention.

### Electronic supplementary material

Below is the link to the electronic supplementary material.

Supplementary Material 1

## Data Availability

Data is provided within the manuscript or supplementary information files; Data available upon contact with corresponding author.

## Abbreviations

AVF: Arterial venous fistular
ADQI: Acute Dialysis Quality Initiative Workgroup
COi: Cardiac output index
EF: Ejection fraction
E/A: Peak early diastolic transmitral flow velocity/peak late diastolic transmitral flow velocity
E/e′: Peak early diastolic transmitral flow velocity/peak early diastolic mitral annular tissue velocity
FAC: Fractional area change
LAVi: Left atrial volume index
IVST: Interventricular septal thickness
LVG: Left ventricular geometry
LVEDVi: Left ventricular end-diastolic volume index
LVESVi: Left ventricular end-systolic volume index
LVPW: Left ventricular posterior wall thickness
LVGLS: Left ventricular global longitudinal strain
LVMI: Left ventricular mass index
MHD: Maintenance hemodialysis
HFpEF: Heart failure with a preserved ejection fraction
HR: Hazard ratio
HF: Heart failure
RVGLS: Right ventricular global longitudinal strain
RWT: Relative wall thickness
TAPSE: Tricuspid annular plane systolic excursion
